# Sitagliptin-induced hemolysis

**DOI:** 10.4103/0253-7613.70405

**Published:** 2010-10

**Authors:** Ragini Bekur, M.V. Nagaraja, K.N. Shivashankara, Weena Stanley

**Affiliations:** Department of Medicine, Kasturba Medical College, Manipal University, Manipal, India

**Keywords:** Dipeptidyl peptidase inhibitor, hemolysis, sitagliptin

## Abstract

Sitagliptin is a newer oral hypoglycemic drug of the dipeptidyl peptidase-IV inhibitor class. It appears to be a promising newer oral hypoglycemic agent. The advantages are the absence of hypoglycemia when used as monotherapy and they cause less gain weight. We report a case of sitagliptin-induced hemolysis, a rare side effect, not reported in the literature. As sitagliptin is widely used in type 2 diabetes mellitus physicians should be aware of the possibility of this rare but potentially serious adverse drug reaction.

## Introduction

Type 2 diabetes (T2DM) is a progressive disorder. In recent years, our understanding of the “entero-insular” axis has lead to the development of new treatments in T2DM such as glucagon-like peptide (GLP) and dipeptidyl peptidase (DPP) IV enzyme inhibition. Sitagliptin, a DPP-IV inhibitor, is a new oral hypoglycemic agent that enhances the body’s own ability to lower the elevated blood glucose and its action is glucose dependent. Headache, sore throat, nausea, arthralgia, myalgia, rash, hives, swelling of the lips, dysphagia, and dyspnea are the common side effects of sitagliptin. We report hemolysis, a rare side effect of this drug.

## Case Report

A 62–year-old patient was known to be with T2DM and hypertension for 15 years. He was treated previously with various sulphonylureas and repaglinide, in addition to ramipril (10 mg), irbesartan (300 mg), and thiazide (12.5mg) for hypertension. His blood sugar was uncontrolled however his hypertension was under control. In the year 2002, he was prescribed rosiglitazone in addition to above drugs, He developed pulmonary edema. Premixed human insulin and metformin were started. In view of his uncontrolled blood sugars and high HbAIC (more than 9%), he was prescribed tab. sitagliptin (100 mg) once daily along with premixed human insulin and metformin. As he had history of adverse drug reactions to rosiglitazone earlier, baseline investigations were done, including liver function tests, before starting sitagliptin, which were normal. After starting sitagliptin, his liver function tests were abnormal, with raised total bilirubin 2.1 mg\dl (normal value <1 mg\dl), direct bilirubin 0.3 mg(normal value <0.6 mg\dl), and indirect bilirubin 1.8 mg\dl, with normal liver enzymes. The drug was stopped as the patient was apprehensive about increasing bilirubin levels. He was readmitted after 1 year, in view of uncontrolled blood sugar and tab sitagliptin was readministered to his premixed human insulin and metformin regimen. His baseline liver function tests showed total bilirubin 1.2 mg, direct bilirubin 0.4 mg, lactate dehydrogenase (LDH) 396 U\L(340-670 U\L), reticulocyte count 0.9%, and Hb 13.5 gm%. After 1 week of treatment, his total bilirubin was to 1.9 mg, direct bilirubin was 0.4 mg, and Hb was 13.5 gm%. The reticulocyte count increased to 4.32%, LDH was 789 U\L, and other liver enzymes were normal. Direct and indirect Coomb’s tests were negative. Peripheral smear showed evidence of polychromasia. The drug was stopped. Bilirubin and reticulocyte count rechecked after 10 days, which showed reduction in total bilirubin of 1.1 mg and reticulocyte count of 0.9%. A diagnosis of sitagliptin-induced hemolysis was made in view of predominant indirect hyperbilirubinemia, raised reticulocyte count, and increased LDH. The causality assessment scales used to interpret the data were Naranjo algorithm score (definite) and WHO probability scale (probable). Severity of adverse drug reaction (ADR) was mild.

## Discussion

T2DM accounts for 90-95% of all diagnosed cases of diabetes. Recent studies have shown that early intervention at pre-diabetic state and beta-cell protection with insulin sensitizers may improve the prognosis of diabetes.[1,2] Any new oral hypoglycemic drug that can improve the control of blood sugar with fewer adverse effects may be welcomed. DPP-IV inhibitor therapy increases the levels of endogenous biologically active incretins, which in turn reduce fasting and postprandial glucose levels, reduce HbA1C values, and improve insulin sensitivity and beta-cell function.[3] Thus they hold considerable therapeutic potential for individuals with T2DM. Treatment with sitagliptin is generally well tolerated. In the controlled clinical studies as both monotherapy and combination therapy with metformin\pioglitazone the overall adverse reactions, hypoglycemia, and discontinuation of therapy due to clinical adverse reactions with sitagliptin were similar to placebo and there were no statistically significant differences in the incidence of either hypoglycemia or predefined gastrointestinal adverse experiences between sitagliptin and placebo groups.[4,5] Due to the glucose-dependent nature of the incretin hormones, insulinotrophic effects do not occur during euglycemia or hypoglycemia. The pooled data from two monotherapies and two combination trials showed that incidence of hypoglycemia was 1.2% and 0.9% for sitagliptin 100 mg and placebo, respectively.[6] Sitagliptin is not as likely to cause hypoglycemia as some other oral hypoglycemic agents; however, care is cautioned while prescribing other drugs that can potentially lower the blood sugar such as probenecid, nonsteroidal anti-inflammatory drugs (NSAIDs), aspirin, sulpha drugs, monoamine oxidase inhibitor (MAOI), or beta blockers.[7] Studies on vildagliptin and sitagliptin have shown to be body weight- neutral in the long term.[8] So far treatment with sitagliptin has not been associated with serious adverse events. Although the emerging safety profile of the DPP-IV inhibitors appears to be excellent, the long-term safety of these agents has not yet been ascertained. The patient in this case developed hemolysis with sitagliptin as evidenced by increase in the total and indirect bilirubin and significant raise in the reticulocyte count and LDH which became normal after stopping the drug. This is the rare side effect noticed, not reported so far. The DPP-IV inhibitors appear to have great potential for the treatment of T2DM, since it is a newly introduced drug, its long-term safety is yet to be established.

## Conclusion

This case is reported to emphasize a rare and unusual adverse reaction to sitagliptin. Even though hemolysis is a rare adverse event of sitagliptin, the frequent use of these drugs makes it important to exercise caution for this potential event. Prevention and early recognition of sitagliptin-induced hemolysis is critical to prevent serious sequelae.
